# An Experimental Study on the Thermal Stability of Mg_2_Si/Ni Interface under Thermal Cycling

**DOI:** 10.3390/ma13143117

**Published:** 2020-07-13

**Authors:** Sung-Jae Joo, Ji Eun Lee, Bong-Seo Kim, Bok-Ki Min

**Affiliations:** Energy Conversion Research Center, Korea Electrotechnology Research Institute, 12 Boolmosan-Ro 10 beon-gil, Changwon 51543, Korea; jieunlee@keri.re.kr (J.E.L.); bskim@keri.re.kr (B.-S.K.); bkmin@keri.re.kr (B.-K.M.)

**Keywords:** thermoelectric material, Mg_2_Si, Ni, contact, interface, temperature cycling, stress-induced voiding

## Abstract

Mg_2_Si is a promising eco-friendly thermoelectric material, and Ni is suited for electrical contact on it. In this study, Bi-doped Mg_2_Si ingots with Ni contacts were fabricated by co-sintering, and thermal stability was investigated by long-time (500 h, 500 cycles) temperature cycling from 25 °C to a peak temperature (*T_h_* = 400 and 450 °C) in N_2_. The as-sintered Ni/Mg_2_Si interfacial region is a multilayer consisting of Mg_3_Bi_2_, a series of Mg_x_Si_y_Ni_z_ ternary compounds (ω, ν, ζ, and η-phases), and MgNi_2_. In the complex microstructure, the MgNi_2_ / η-phase interface was vulnerable to stress-induced voiding at *T_h_* = 450 °C, which arises from the mismatch of the thermal expansion coefficients. Interfacial voiding was avoided by adding 10 mol% Ag in Ni, which is probably due to the suppression of vacancy migration by the Ag-containing 2nd phase formation at the MgNi_2_/η-phase interface.

## 1. Introduction

In recent years, the regulations on greenhouse gases are being strengthened continuously, and the recovery of waste heat is becoming increasingly important as a viable means of raising the energy efficiency of fossil fuels. Thermoelectric generation (TEG) is an energy conversion technique based entirely on solid-state devices utilizing the Seebeck effect, and has many attractive features such as silent operation, long-term reliability, compactness, and scalability. However, along with the potential advantages, thermoelectric technology also has a few drawbacks to be resolved, among which the cost as well as the non-toxicity of the materials are very important requirements. In this regard, Mg_2_Si is a very attractive material because it is inherently composed of non-toxic earth-abundant elements, which also guarantees a low material cost. Furthermore, thermoelectric performances are also excellent when making solid solutions with Mg_2_Sn, which has pushed up the dimensionless figure of merit *ZT* up to about 1.3 in Sb-doped Mg_2_Si_0.3_Sn_0.7_ by utilizing the band convergence and alloy-scattering effects [1,2,3]. Thus, Mg_2_Si-based materials are surely very promising for future thermoelectric applications.

Meanwhile, a robust electrical contact is another prerequisite for TEG module development. The ideal contact should conduct heat and electricity with low resistances, and the interface with the TE material should be highly stable during the operation lifetime of the module. To date, Ni has been studied most widely for contacts as well as intermediate diffusion barriers for Mg_2_Si due to the low electrical resistivity (69.3 nΩ·m at 20 °C), a high thermal conductivity (90.9 Wm^−1^K^−1^), and a comparable thermal expansion coefficient (TEC) of 13.4 × 10^−6^ at 25 °C with that of Mg_2_Si (13.1 × 10^−6^ at 25 °C [4]). Referring to previous studies, the Ni/Mg_2_Si combination was reported to have contact resistances lower than 10 μΩ·cm^2^ [5]. Long-time isothermal annealing tests, such as annealing at 450 °C for 600 h [6] or at 550 °C for 168 h [7], also demonstrated good thermal stability. However, isothermal annealing test is not enough to confirm the reliability, and temperature cycling (TC) is more appropriate to examine the robustness of the Ni/Mg_2_Si interface for real situations. To the authors’ knowledge, there has been no reports on the long-time TC test of the Ni/Mg_2_Si heterointerface, which is the motivation of this study.

In this study, Ni was co-sintered with Mg_2_Si to fabricate Ni/Mg_2_Si/Ni bulk samples, and the long-time stability of the Ni/Mg_2_Si interfaces was studied by TC. The main goal of the study was to identify the failure mechanism of a Mg_2_Si/Ni system under repetitive thermal stress, as well as to define the temperature regime for long-term safe operation. The interface compounds and evolution of the microstructure before- and after TC were investigated, and a solution to enhance the interface reliability was devised.

## 2. Materials and Methods

### 2.1. Materials and Mg_2_Si/Ni co-sintering

Highly pure powders of Mg (>99.99 wt.%), Si (>99.9999 wt.%), and Bi (>99.999 wt.%), were used to fabricate the Bi-doped Mg_2_Si samples. First, source powders of Mg and Si were mixed with approximate molar ratio of 2.08:1 so that the inevitable loss of volatile Mg during the synthesis is compensated. A total of 0.05 mol % Bi was also added for n-type doping, and then the mixed powders were cold-pressed, sealed in an Ar-filled quartz tube, and heated for 24 h at 700 °C for solid state reaction. The reacted material was then crushed manually and milled by using a planetary ball miller at 300 RPM (PM-100, Retsch, Inc., Haan, Germany). After ball milling, the powders were sieved to filter out large particles (diameter > 45 μm), and loaded into a graphite mold (diameter = 12.7 mm) where Ni powders (>99.9 wt.% purity, diameter <3.0 μm) were loaded first at the bottom. Ni powders were also carefully loaded on top of the Mg_2_Si powders to form a Ni/Mg_2_Si/Ni structure, and the co-sintering was implemented by applying 150 MPa pressure at 800 °C for 60 min using a hot press (HP) system. The sintered ingots had cylindrical shapes with the diameter and height of 12.7 mm and about 12 mm, respectively, and the thickness of the Ni layers on both sides of the Mg_2_Si was about 300 μm. In some experiments, mixed powders of Ni and Ag with a molar ratio of 9:1 were used for co-sintering to examine the effect of Ag addition on the interface morphology and thermomechanical stability, and the purity and the average particle size of the Ag powder were 99.9% and about 50 nm, respectively.

### 2.2. Temperature Cycling and Characterization

After sintering, the ingots were diced into smaller pieces for thermal tests. TC was implemented in a furnace equipped with a movable sample holder, which enables periodic movements of the samples into and out of the hot zone as programmed. A thermocouple attached to the holder measured the real-time temperature of the samples. After loading the samples, the furnace was evacuated to a base pressure of about 5 mTorr, and then filled with high purity N_2_ (>99.999%) to 1 atm. This procedure of evacuation and N_2_ purging was repeated several times to minimize the O_2_ partial pressure, and thermal tests were initiated under 1 atm N_2_ atmosphere with continuous replenishment of fresh N_2_ into the furnace. One cycle of the TC tests consisted of moving the samples into the hot zone of the furnace where the temperature was fixed at *T_h_* (400 and 450 °C), staying for 30 min, moving out of the hot zone, and natural cooling for 30 min in the unheated zone. This was repeated 500 times without stopping, which took about 500 h for the whole process.

After TC, the samples were grinded with SiC abrasives and polished with fine colloidal silica suspension (0.04 μm), after which the cross sections were investigated using a field emission scanning electron microscope (SEM) equipped with an energy dispersive X-ray spectroscopy (EDS) system (Tescan, MIRA II, Brno, Czechia). Multi-purpose thin-film X-ray diffractometer (XRD, Rigaku D/MAX-2500, Tokyo, Japan) was also utilized to identify the phases in the samples. The specific contact resistance (SCR) was measured by a potential Seebeck microprobe (PSM) equipment (Panco, Muelheim Kaerlich, Germany), where the electrical resistivity and the Seebeck coefficient (a measure of the magnitude of an induced voltage within a material versus a temperature difference applied across that material) of a material are simultaneously measured with a spatial resolution of tens of micrometers [8].

## 3. Results and Discussion

### 3.1. As-Sintered Mg_2_Si/Ni Interfaces

**Figure 1:**
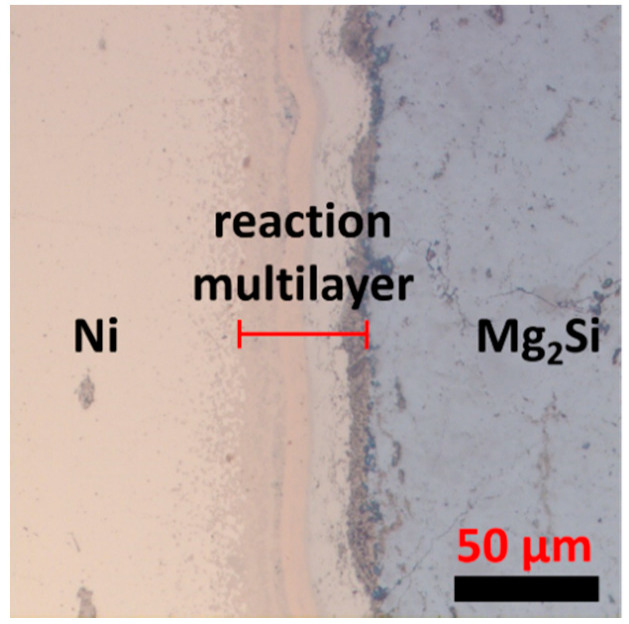
A cross-sectional optical microscope image of the Ni/Mg_2_Si interfacial region of an as-sintered sample.

**Figure 2:**
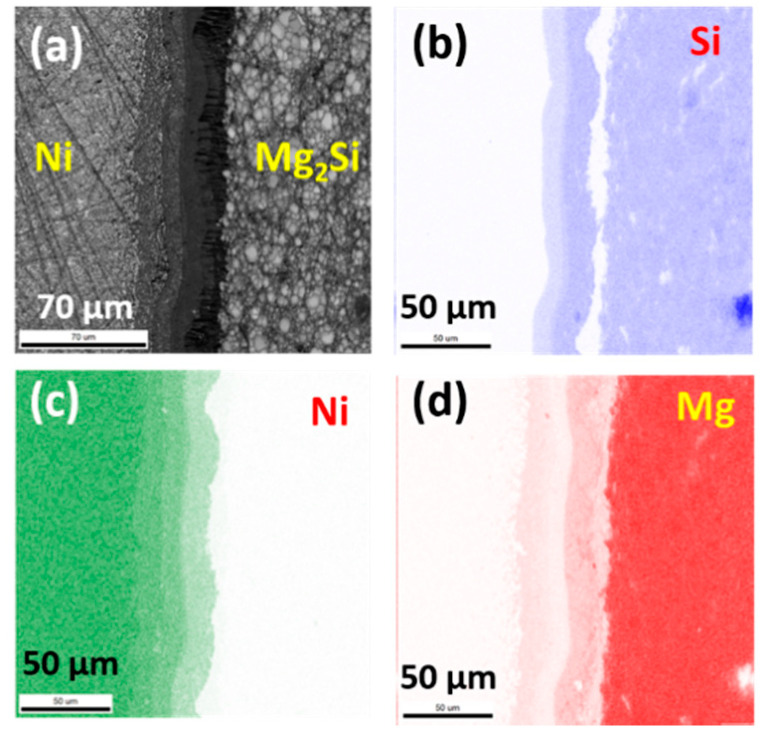
(**a**) A cross-sectional SEM image of an as-sintered sample, and the corresponding 2-dimensional element maps of (**b**) Si, (**c**) Ni, and (**d**) Mg, respectively.

Figure 1 shows an optical microscope image of the cross section of an as-sintered sample, where the multiple reaction layers are clearly visible at the Ni/Mg_2_Si interface. The total thickness of the reaction layers was about 50 μm, and the multilayer was continuous across the whole interface. Cracks or voids were not observed at the interface of the as-sintered samples. EDS was employed to identify the multilayer, and Figure 2 shows the two-dimensional maps of the major elements; Si, Ni, and Mg, respectively, and an SEM image. The clear contrast in the maps indicates that each layer has distinctly different stoichiometry, and from Figure 2 we can also see that multiple layers of Mg_x_Si_y_Ni_z_ ternary compounds exist at the interface.

**Figure 3:**
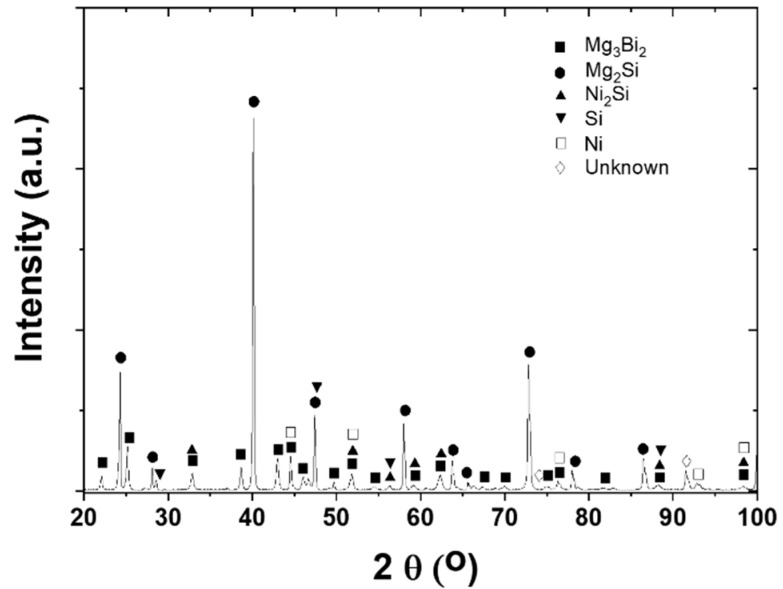
XRD patterns from an as-sintered Ni/Mg_2_Si sample.

XRD was employed to identify the phases in the interfacial reaction layers, and Figure 3 shows the result. Naturally, the most prominent peaks coincide with those of Mg_2_Si, while many small peaks suggest the possible existence of Mg_3_Bi_2_, Ni_2_Si, and Si. A few peaks could not be clearly identified as marked in Figure 3, and the overlap of peak positions from different phases made it difficult to interpret the XRD patterns unambiguously. Unfortunately, any Mg_x_Si_y_Ni_z_ ternary compounds were not identified from the XRD patterns according to the material database, which is inconsistent with the EDS result of Figure 2. Basically, the weak signal from the interfacial layers may not be easily observable in the XRD patterns, so more detailed analysis of the interfacial microstructure was implemented using SEM and EDS.

**Figure 4:**
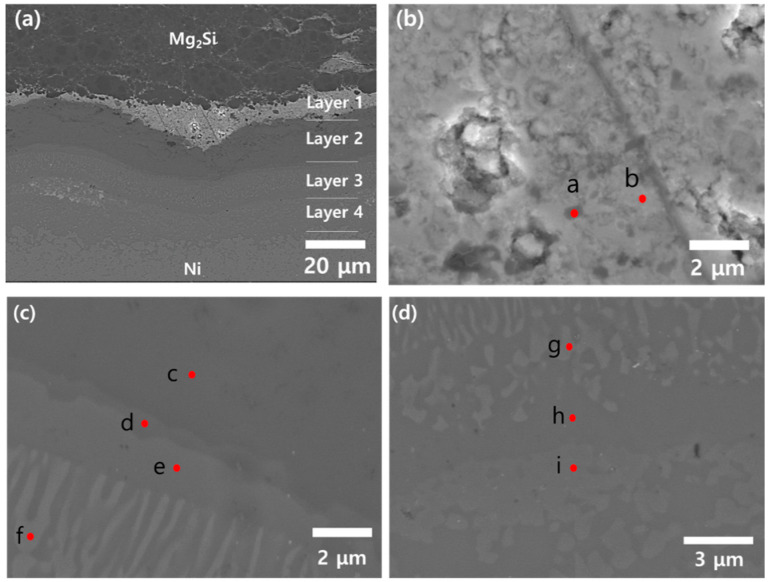
(**a**) A cross-sectional SEM image of an as-sintered sample showing 4 distinct layers, and magnified images of (**b**) the Layer 1, (**c**) the interface between the Layer 2 and 3, and (**d**) the interface between the Layer 3 and 4, respectively.

Figure 4a shows a typical SEM image of the interfacial region between the Bi-doped Mg_2_Si and Ni just after co-sintering, and four distinct layers are clearly visible, which were marked as Layer 1, 2, 3, and 4, respectively. First, Figure 4 b shows the magnified image of the Layer 1, and the quantitative EDS results on the points a and b are summarized in Table 1, which confirms that Mg, Bi, and O are the dominant elements in Layer 1. Referring to the XRD result of Figure 3, it is appropriate to conclude that Layer 1 is composed of Mg_3_Bi_2_ and oxide phases such as MgO and Bi_2_O_3_. It is noted that Bi was originally added as an n-type dopant, but a considerable amount of Bi was consumed for Mg_3_Bi_2_ formation at the interfacial region. According to a previous report, thermodynamic calculations and experimental data show that Mg_3_Bi_2_ formation is a thermodynamically favorable process with a large negative Gibbs free energy (about −28 kJ/mole) at the temperatures of 702 °C and 850 °C [9], which are close to the sintering temperature (800 °C) of this study.

Next, Figure 4c shows a magnified image of the interface between the Layer 2 and 3. The spot c belongs to the homogeneous Layer 2, and the major constituent is Mg, Si, and Ni, as shown in Table 1. Therefore, the Layer 2 is a Mg_x_Si_y_Ni_z_ ternary layer. According to the previous studies there are many different Mg_x_Si_y_Ni_z_ ternary phases [10,11,12], and clearly identifying a specific phase from a full list of ternary compounds is not straightforward. Nevertheless, the composition ratio at the point c suggests that the Layer 2 is the ω-phase, whose approximate composition and suggested stoichiometry are Mg_33_Si_37_Ni_30_ and (Mg_0.52_Ni_0.48_)_7_Si_4_, respectively [10]. Moving downward, the point d represents a thin layer adjacent to ω-phase, and the quantitative EDS analysis suggests that ν-phase (Mg_11_Si_10_Ni_12_) is highly probable. The points e and f belong to the Layer 3 and have slightly different contrast in the SEM image. However, EDS results suggest that both spots are ζ-phase (Mg_3_Si_7_Ni_10_).

Figure 4d shows the interfacial region of the Layer 3 and 4, and it is shown that isolated regions of Ni_2_Si (spot g) are surrounded by η-phase (spot h, Mg_20_Si_24_Ni_56_). Adjacent to the point h, the phase at the point i was confirmed to be MgNi_2_, and the Layer 4 was identified to be a mixed zone of MgNi_2_ and Ni, without any Mg_x_Si_y_Ni_z_ ternary phase. Finally, the region below the Layer 4 in Figure 4a is Ni.

Summarizing the results, the as-sintered Ni/Bi-doped Mg_2_Si contacts were composed of intermediate reaction layers including Mg_3_Bi_2_, Ni_2_Si, Mg_x_Si_y_Ni_z_ ternary compounds (ω, ν, ζ, η phases), and MgNi_2_. Basically, this feature was reproduced identically in every as-sintered sample. It is noted that four different Mg_x_Si_y_Ni_z_ ternary phases were observed simultaneously. Referring to a previous study, Iida et al. reported that ω- and η-phases were observed at the Ni/(Al, Sb)-doped Mg_2_Si, but formation of the ν- and the ζ-phases was not noted [13].

Although interfacial reaction is inevitable and indispensable for bonding strength and conductivity, intermediate layers with complex structures may result in unexpected effects to degrade a structural reliability, which is demonstrated in the next section.

### 3.2. Mg_2_Si/Ni Interfaces after Temperature Cycling

**Figure 5:**
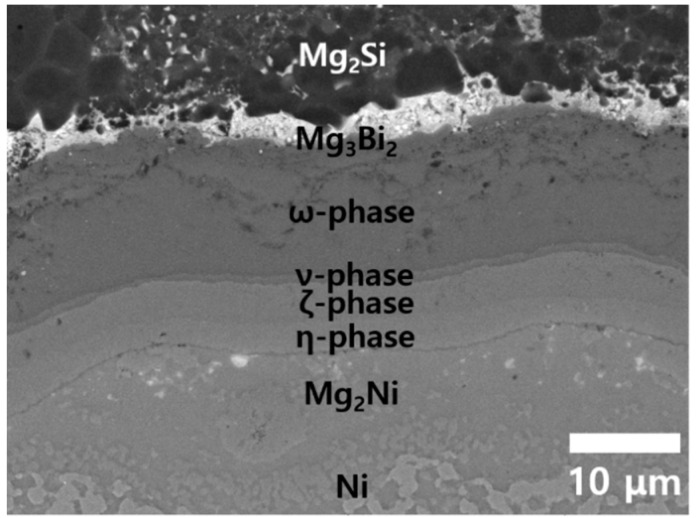
A cross-sectional SEM image after TC from 25 °C to the peak temperature T_h_ = 400 °C for 500 times.

Figure 5 shows a cross-sectional SEM image of a Ni/Mg_2_Si sample after TC for 500 times from 25 to 400 °C. The basic features of the interfacial reaction layers from Mg_3_Bi_2_ to Mg_2_Ni, including a series of ternary phases ω, ν, ζ, and η, are the same after long-time TC, and the thickness of each layer also did not show noticeable change. Any structural imperfections, such as voids and cracks, are not observed at the interfaces, indicating that the Ni/Mg_2_Si interface has thermomechanical stability against TC with T_h_ ≤ 400 °C.

**Figure 6:**
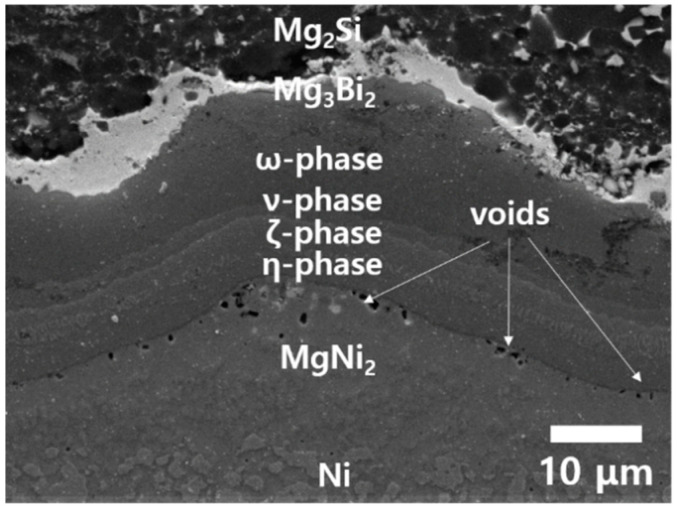
A cross-sectional SEM image after TC from 25 °C to the peak temperature T_h_ = 450 °C for 500 times, showing many voids at the η-phase/MgNi_2_ interface.

However, when T_h_ was raised to 450 °C during the TC, a different result was obtained, as shown in Figure 6. Many voids were produced along the η-phase/MgNi_2_ interface, and this means that Ni/Mg_2_Si interface is not structurally reliable when exposed repeatedly to temperatures above 450 °C over a long time, mainly due to the weakness of the η-phase/MgNi_2_ interface. The void formation shown in Figure 6 is regarded to be a typical case of stress-induced voiding (SIV), and the driving force arises from the thermal stress σ_t_ due to the difference of the TECs

σ_t_ = E × (α_1_ – α_2_) × ΔT (1)

where E, α, ΔT is the elastic modulus, TEC, and temperature difference, respectively. It is well known that the thermal stress activates vacancy diffusion at the heterointerface, and SIV has been a critical problem in the multi-level metallization process for semiconductor microchip fabrication [14,15,16,17]. The SIV is expected to be more severe in mid-temperature Mg_2_Si thermoelectric modules because the operation temperature as well as the temperature swing are much higher. Nonetheless, we suppose that this is the first relevant report to the best of the authors’ knowledge. For now, the critical σ_t_ above which SIV begins cannot be estimated because the E and α of MgNi_2_ and the η-phase are unknown, which requires further study.

**Figure 7:**
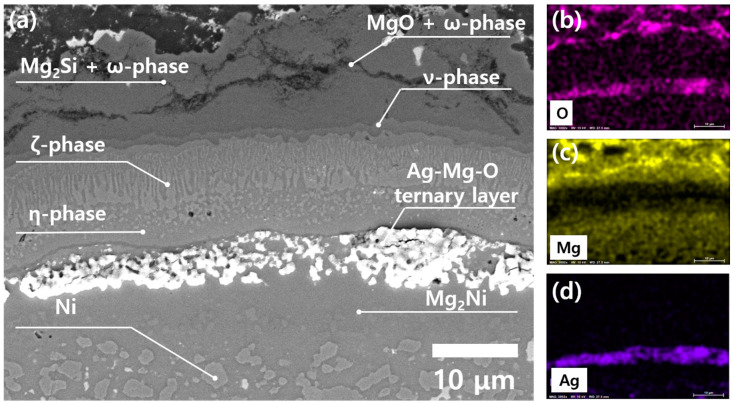
(**a**) A cross-sectional SEM image of a sample containing 10 mol% Ag in Ni, showing Ag-containing agglomerates at the η-phase/MgNi_2_ interface. A 500 times TC from 25 to 450 °C was applied to the sample. The EDS results of (**b**), (**c**), and (**d**) show the distribution of elements; O, Mg, Ag, in the same area, respectively.

Meanwhile, a solution to mitigate the SIV at Ni/Mg_2_Si system was devised. Figure 7 shows the cross-section of a sample containing 10 mol% Ag in Ni, which has undergone 500 times TC of T_h_ = 450 °C. The reason that Ag was mixed with Ni was based on the initial guess that Ag can form the 2nd phase within the Ni matrix without forming intermetallic compounds with Ni, which would possibly retard the vacancy diffusion. Indeed, Figure 7 confirms that the 2nd phase agglomerates containing Ag, Mg, and O have formed along the η-phase/MgNi_2_ interface, and the SIV was not observed. Meanwhile, the dark region in the middle of {Mg_2_Si + ω-phase} layer is not a crack, but a mixed zone of MgO and the ω-phase, as marked in Figure 7a. Although more thorough study based on experimental data is required to draw a definite conclusion, we think that suppressing SIV by 2nd phase inclusions would be a plausible idea to enhance the structural reliability of a Ni/Mg_2_Si heterointerface.

### 3.3. PSM Analysis of Mg_2_Si/Ni Interfaces

**Figure 8:**
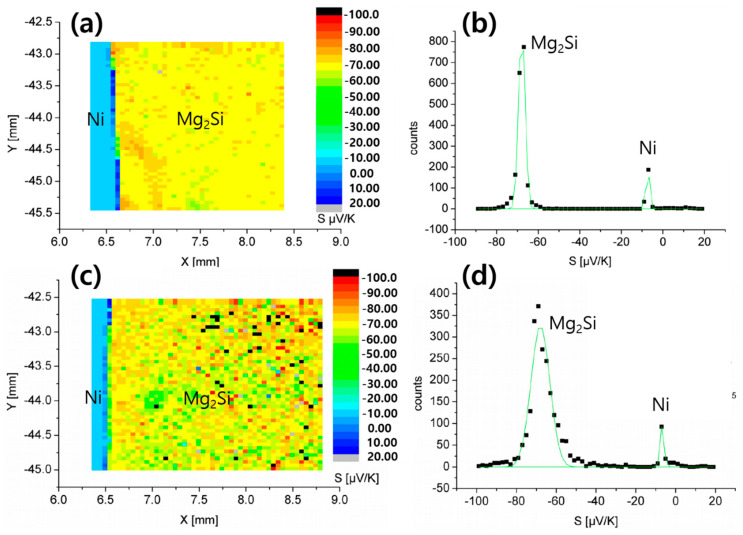
2-dimensional maps of the Seebeck coefficient S and the corresponding S-value distribution profiles. (**a**) and (**b**): an as-sintered sample, (**c**) and (**d**) a TC-applied sample at T_h_ = 400 °C, 500 cycles.

Figure 8 shows the 2-dimensional maps of Seebeck coefficients S and corresponding S-value distribution profiles near the Ni/Mg_2_Si interfaces before and after 500-times TC (T_h_ = 400 °C), which were obtained by PSM analysis. The distribution profiles shown in Figure 8b,d have two peaks originating from Mg_2_Si and Ni, respectively, among which that of Ni is naturally close to zero. The average S of Mg_2_Si changed little after TC, from−67.88 to−67.98 μV/K, but the peak became broader as confirmed from the increase in the full width at half maximum (FWHM) from 3.09 to 9.43 μV/K. The change in local composition by Mg outdiffusion as well as oxidation at the interface region may have contributed to the FWHM increase.

The SCR was also measured by PSM, and Table 2 summarizes the results. Contrary to Seebeck coefficient, the mean value of SCR decreased by about 73% after TC, and the standard deviation was also reduced. The reason why the SCR has decreased considerably after TC remains to be discovered, and, for now, we suppose that many interfacial mid-gap states impeding electron conduction would have been annealed out during the heat process. The mean value of the SCR was about 11.4 μΩ⋅cm^2^ after 500 times TC with T_h_ = 400 °C, which is comparable with the previous result of SCR < 10 μΩ⋅cm^2^ obtained by de Boor et al. [5,7]. Therefore, a Ni contact on Mg_2_Si shows a superior performance in terms of contact resistance even after TC.

## 4. Conclusions

In this study, Ni contacts on Mg_2_Si were formed by co-sintering, and the interfacial microstructure and the thermal stability of the Ni/Mg_2_Si heterointerfaces were investigated. The interfacial region of the as-sintered Ni/Mg_2_Si samples was composed of reaction multilayers including Mg_3_Bi_2_, Ni_2_Si, Mg_x_Si_y_Ni_z_ ternary compounds (ω, ν, ζ, and η phases), and MgNi_2_. Long-term TC was applied to samples to investigate the thermomechanical reliability, and the Ni/Mg_2_Si heterointerface was confirmed to be robust below 400 °C without any structural imperfections. However, above the peak temperature of 450 °C, SIV was initiated at the η-phase/MgNi_2_ interface, which was found to be the weakest part in the Ni/Mg_2_Si contacts. Accordingly, a smart solution to mitigate the TEC difference at the η-phase/MgNi_2_ interface would be required for operation at higher temperatures. By adding 10 mol% Ag in Ni before co-sintering, the SIV was suppressed at 450 °C. The SCR of the Ni/Mg_2_Si heterointerface was about 11.4 μΩ⋅cm^2^ after 500 times TC with *T_h_* = 400 °C, which is low enough for practical application.

## Figures and Tables

**Table 1:** Quantitative EDS results on the spots in Figure 4.

Spot	Mg (at.%)	Si (at.%)	Ni (at.%)	Bi (at.%)	O (at.%)	C (at.%)	Candidate Phase
a	41.12	-	1.81	16.57	33.26	7.24	Mg_3_Bi_2_ + oxides
b	30.95	-	2.21	18.63	36.06	12.15	Mg_3_Bi_2_ + oxides
c	20.75	22.67	18.04	-	3.27	35.26	(Mg_0.52_Ni_0.48_)_7_Si_4_ (ω-phase)
d	17.21	21.32	26.40	-	3.08	31.99	Mg_11_Si_10_Ni_12_(ν-phase)
e	12.84	22.57	30.41	-	3.71	30.47	Mg_3_Si_7_Ni_10_ (ζ-phase)
f	9.97	18.23	36.93	-	2.98	31.89	
g	2.56	20.28	39.46	-	2.97	34.74	Ni_2_Si
h	14.20	15.93	35.49	-	2.66	31.72	Mg_20_Si_24_Ni_56_(η-phase)
i	16.85	-	45.20	-	2.89	35.07	MgNi_2_, Ni

**Table 2:** SCRs of the sample before- and after TC measured by PSM method.

As-Sintered Sample	Temperature Cycled Sample (T_h_ = 400 °C, 500 Cycles)
43.4 ± 24.5 μΩ·cm^2^	11.4 ± 5.0 μΩ·cm^2^
